# Relationships among the triglyceride-glucose index, its changes, and the development of metabolically obese normal weight are strengthened by increased visceral fat area

**DOI:** 10.3389/fnut.2025.1642725

**Published:** 2025-09-16

**Authors:** Jianan Wang, Jingjing Wang, Yutian Lei, Qin Yuan, Xueyao Yin, Fenping Zheng

**Affiliations:** ^1^Department of Endocrinology, The Affiliated Sir Run Run Shaw Hospital, College of Medicine, Zhejiang University, Hangzhou, China; ^2^Phase I Clinical Trial Center, The Affiliated Zhejiang Hospital, College of Medicine, Zhejiang University, Hangzhou, China; ^3^Department of Endocrinology, Longyou Branch Hospital of the Affiliated Sir Run Run Shaw Hospital, College of Medicine, Zhejiang University, Quzhou, China

**Keywords:** triglyceride-glucose index, changes in triglyceride-glucose index, metabolically normal, normal weight, metabolically obese normal weight, visceral fat area

## Abstract

**Background:**

Metabolically obese normal weight (MONW), obesity with a normal body mass index, is often neglected due to the seemingly normal weight but has a high risk of metabolic diseases. This study aimed to assess associations among the triglyceride-glucose (TyG) index, its changes (∆TyG index), and metabolically normal, normal weight (MNNW) to MONW transition via a population-based cohort study.

**Methods:**

Participants aged 40–65 years in 15 Chinese communities were recruited. A total of 530 participants [mean age: 53.00 (48.00–58.00) years; 346 (65.3%) female participants] with complete data on anthropometry, metabolic indicators, abdominal visceral fat area (VFA), and a normal body mass index (BMI) (18.5 ≤ BMI < 24) were included in the cross-sectional analysis. In total, 253 participants [mean age: 53.00 (48.00–58.00) years; 171 (67.6%) female participants] who had follow-up and maintained a normal BMI were included in the follow-up analysis. MONW was defined as two or more abnormal metabolic components but with a normal BMI. Multivariate logistic regression, Cox proportional hazards regression, and restricted cubic spline regression were used to assess associations among the TyG index, ∆TyG index, and MONW-MONW and MNNW-MONW transition.

**Results:**

An incrementally higher risk of MONW was observed with the increasing TyG index quartiles in the cross-sectional analysis. The adjusted odds ratio (OR) in the TyG index quartile 4 was 31.81 (95% CI 11.47–88.20) for MONW, and there was a significant MONW risk for TyG >9.04. A positive linear association between the ∆TyG index (*p* trend = 0.001) instead of the TyG index (*p* for non-linearity = 0.034) and MNNW-MONW transition was observed. The adjusted hazard ratio (HR) in the TyG index quartile 3 was 2.35 (95% CI 1.02–5.41), and in the ∆TyG index quartile 4 was 3.60 (95% CI 1.48–8.79) for MNNW-MONW transition. Subgroup analyses revealed the correlations among the TyG index, ∆TyG index, and MONW-MONW and MNNW-MONW transition were more evident in individuals with larger VFA, and similar results were obtained in sensitivity analysis.

**Conclusion:**

The elevated TyG index and ∆TyG index were associated with higher risks of MONW-MONW and MNNW-MONW transition, and these associations were strengthened by VFA. In addition, the ∆TyG index may be a better indicator for predicting MNNW-MONW transition.

## Introduction

Metabolically obese normal weight (MONW), a special type of obesity with a normal body mass index (BMI) but symptoms of metabolic dysfunction, adversely affects the lipid profile and blood pressure, intensifies inflammatory, thrombotic processes, and oxidative stress, and has a higher risk of type 2 diabetes, cardiovascular disease (CVD) and all-causes of death compared to metabolically normal, normal weight (MNNW) (1–3). However, surveillance of metabolic indicators and early intervention measures is often neglected in patients with MONW due to the seemingly normal weight. Therefore, early identification of MONW, as well as timely monitoring MNNW-MONW transition via simple and accurate markers could mitigate the metabolic and cardiovascular risks. Nevertheless, literature is limited on MNNW-MONW trajectory; thus, analyzing the risk factors underlying MNNW-MONW transition should be the focus of further research (4–6).

In recent years, the triglyceride-glucose (TyG) index, a composite index composed of fasting triglyceride and fasting glucose, has been proven to be a simple and reliable surrogate biomarker of insulin resistance, even superior to the homeostasis model of assessment-insulin resistance for predicting CVD in the large-scale population (7–10). Furthermore, the TyG index is closely associated with the increased amounts of visceral fat, insulin resistance, and hyperinsulinemia, which are the key parts of the complex and still insufficiently recognized pathogenesis of MONW (11, 12). Therefore, the TyG index and its changes might be effective biomarkers for timely identifying, monitoring, and intervening MNNW-MONW transition and the accompanying CVD risk. This study aimed to explore the role of the TyG index and its changes in the incidence of MONW-MONW and MNNW-MONW transition based on a cohort study in 15 Chinese communities.

## Materials and methods

### Study participants

**Figure 1:**
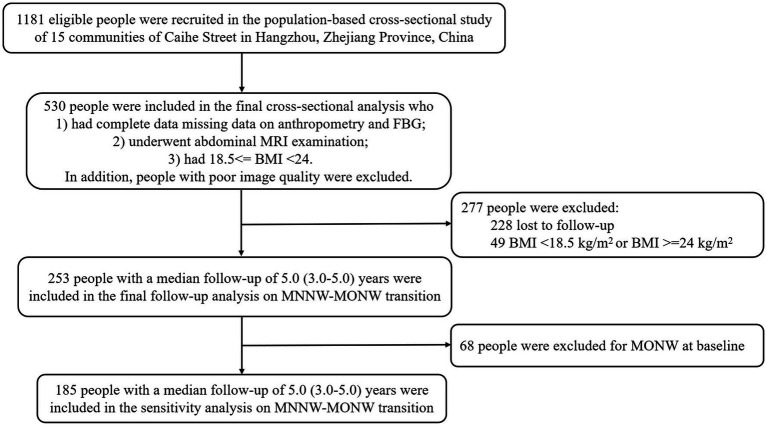
Flowchart of study participant enrollment. FBG, fasting blood glucose; MRI, magnetic resonance imaging; BMI, body mass index; MNNW, metabolically normal, normal weight; MONW, metabolically obese, normal weight.

The population-based cohort study recruited individuals aged 40 to 65 years with at least 5 years of residency on the basis of the stratified cluster random sampling method from 15 communities of Caihe Street in Hangzhou, Zhejiang Province, China, while excluding following participants: (1) previous cardiovascular events, (2) oral or intravenous corticosteroids, (3) cirrhosis and ascites, (4) known hyperthyroidism or hypothyroidism, (5) malignant tumor, (6) severe disability or mental illness, and (7) pregnant and lactating women, and the baseline investigation was performed between March and May 2010, while the follow-up was carried out in 2013 and 2015, as documented previously (13). Finally, a total of 1,181 Chinese Han participants were enrolled in the baseline investigation. The current study was a further analysis of the above population-based cohort study. The inclusion criteria for the baseline cross-sectional analysis of the current study were as follows: (1) had complete data on anthropometry and fasting blood glucose (FBG), (2) underwent abdominal magnetic resonance imaging (MRI) examination, and (3) had 18.5 ≤ BMI < 24 (normal BMI range was based on the criteria of the Working Group on Obesity in China (14)). Participants with poor image quality due to motion artifacts were excluded. In the follow-up analysis of MNNW-MONW transition, in addition to the inclusion and exclusion criteria in the cross-sectional analysis, we further excluded participants who (1) were lost to follow-up and (2) had a BMI <18.5 or ≥24 at the follow-up (Figure 1).

### Data collection

Demographic characteristics, medical history, and lifestyle risk factors were obtained via rigorous standardized questionnaires and through quality control systems by trained medical investigators: age, sex, medical history of diabetes mellitus (DM) and hypertension, smoking status, alcohol consumption, and physical activity. Smoking status was classified as non-smoker, ex-smoker, or current smoker. Alcohol consumption was categorized as no drinking (none), less than three times a month (mild), or three or more times a month (heavy). Exercise data were collected via the International Physical Activity Questionnaire Short Form (IPAQ-SF), and physical activity was categorized as low, moderate, or high levels according to the criteria of the IPAQ Research Committee (15).

Basic anthropometric data, including weight, height, waist circumference, and blood pressure, were measured via established techniques by trained staff, as described previously (16). In brief, weight was measured in a fasting state without shoes and wearing light clothes. Height was measured when participants took off the shoes and stood upright. Waist circumference was measured by a tape measure at the midpoint between the lower border of the rib cage and the iliac crest. Blood pressure was measured in triplicate using a mercury sphygmomanometer in Sir Run Run Shaw Hospital, and the average of the three measurements was recorded. After at least 10 h of overnight fasting, whole-blood and serum samples were collected from all participants. Blood samples were measured in the central laboratory of Sir Run Run Shaw Hospital for the 75-g oral glucose tolerance test (OGTT). FBG, 2-h post-load plasma glucose (2hPG), total cholesterol, triglyceride, high-density lipoprotein cholesterol (HDL-C), and low-density lipoprotein cholesterol (LDL-C) were measured with enzymatic method by auto analyzer (Aeroset, Chicago, IL, United States).

Abdominal subcutaneous fat area (SFA) and visceral fat area (VFA) at the umbilical level between L4 and L5 were scanned with MRI examination using a Signa 1.5 T MRI device equipped with an abdominal coil (SMT-100; Shimadzu Corp., Kyoto, Japan) and analyzed via SliceOmatic image analysis software (version 5) on the basis of 2-D pixels in DICOM images fitting the “adipose shading threshold.”

### Definition and diagnosis

DM was defined as self-reported DM, any treatment for DM, or those newly diagnosed based on OGTT in this survey. Hypertension was defined as self-reported hypertension, any treatment for hypertension, or systolic blood pressure (SBP) ≥140 mmHg, or diastolic blood pressure (DBP) ≥90 mmHg in this survey. BMI was calculated as weight (kg) divided by the square of height (m^2^). eGFR was calculated using the modified Modification of Diet in Renal Disease equation: eGFR = 175 × Scr (mg/dL) − 1.234 × Age^−0.179^ × 1 (male) or 175 × Scr (mg/dL) − 1.234 × Age^−0.179^ × 0.79 (female) (17). Remnant cholesterol concentration was calculated as the total cholesterol concentration minus the LDL-C concentration minus the HDL-C concentration (18). The TyG index was calculated as ln[triglyceride (mg/dL) × FBG (mg/dL)/2] (19). The ∆TyG index was defined as (the TyG index at follow-up—the TyG index at baseline)/the TyG index at baseline × 100%.

General obesity was defined by BMI according to the criteria of the Working Group on Obesity in China (14). Obesity was defined as BMI ≥28, overweight as 24 ≤ BMI < 28, and normal weight as 18.5 ≤ BMI < 24. Metabolically abnormal components included (1) elevated triglyceride (≥1.7 mmol/L) or treatment with lipid-lowering therapy; (2) low HDL-C (male participants <1.04 mmol/L, female participants <1.29 mmol/L); (3) elevated SBP (≥130 mmHg) or DBP (≥85 mmHg), and (or) confirmed hypertension and (or) treatment with antihypertensive therapy; (4) FBG ≥110 mg/dL or 2hPG ≥140 mg/dL, and (or) diagnosis of DM and (or) treatment with antidiabetic therapy (20, 21). Metabolic obesity was defined as two or more abnormal components, while metabolic normality was defined as ≤1 abnormal component (22, 23). The transitions between MNNW and MONW were categorized into four groups: MNNW-MNNW was defined as MNNW at baseline and throughout the follow-up, MNNW-MONW as MNNW at baseline and MONW at any point during the follow-up, MONW-MONW as MONW at both baseline and the last follow-up, and MONW-MNNW as MONW at baseline and MNNW throughout the follow-up.

### Statistical analysis

Baseline characteristic data were expressed as mean ± standard deviation (SD) for continuous variables with the normal distribution or median (25th–75th percentiles) for continuous variables without the normal distribution and as number (percentage or frequency) for categorical variables. Participant characteristics were stratified by the baseline TyG index quartiles, and comparisons between groups used the *χ*^2^ test for categorical variables, or one-way analysis of variance test (ANOVA) or Kruskal–Wallis test as appropriate for continuous variables. Multivariate logistic regression models were used to assess odds ratios (ORs) and 95% confidence intervals (CIs) for the associations between the TyG index quartiles and the incidence of MONW in the baseline cross-sectional analysis. The associations between the TyG index quartiles, the ∆TyG index quartiles, and the risk of MNNW-MONW transition in the follow-up analysis were investigated using quartile 1 as the reference category via Cox proportional hazards regression models, and the results were reported as hazard ratios (HRs) with 95% CIs. Collinearity was identified by variance inflation factor (VIF) >10. Three regression models were constructed: model 1 was unadjusted; model 2 was adjusted for age and sex; and model 3 was adjusted for age, sex, BMI, waist circumference, VFA, body fat content, remnant cholesterol, smoking status, alcohol consumption, physical activity, hypertension, and DM (All VIFs were less than 10).

Subgroup analyses and interaction testing were also performed stratified by age, sex, remnant cholesterol, and VFA at baseline to explore the potential modifying effects on the basis of likelihood ratio tests. Age and sex were classified into two groups: <55 and ≥55 years, and male and female groups, respectively; remnant cholesterol and VFA were categorized into four groups according to quartiles. Considering the small amount of the study sample size, in these subgroup analyses, the ORs (95% CI) of the association between the TyG index and MONW, and the HRs (95% CI) of the associations between the TyG index, ∆TyG index, and MNNW-MONW transition were based on the analysis of the TyG index and the ∆TyG index as continuous variables.

We used restricted cubic spline (RCS) regression with five knots at the 5th, 35th, 50th, 65th, and 95th centiles (reference is the 5th percentile) to test the potential non-linearity and flexibly model the association of the TyG index with MONW in the cross-sectional analysis and the TyG and ∆TyG index with MNNW-MONW transition in the follow-up analysis. In the spline models, age, sex, BMI, waist circumference, VFA, body fat content, remnant cholesterol, smoking status, alcohol consumption, physical activity, hypertension, and DM were adjusted.

To minimize the possible influence of pre-existing MONW on the associations between the TyG index quartiles, ∆TyG index quartiles, and the risk of MNNW-MONW transition, we also performed sensitivity analysis excluding individuals who were MONW at baseline. All statistical analyses were performed via SPSS Statistics 26.0 (IBM Corp., Armonk, NY, United States) and R software.^1^ Statistical significance was defined as a two-tailed *p* < 0.05.

## Results

### Cross-sectional analysis

A total of 530 eligible participants were included in the final baseline cross-sectional analysis, with 184 male and 346 female participants. Participant characteristics stratified by the TyG index quartiles are presented in Table 1. Participants in the higher quartile (quartile 4) of the TyG index were more likely to be male and had a less favorable metabolic profile than those in the lower quartiles. Participants in quartile 4 exhibited higher levels of BMI, waist circumference, blood pressure, FBG, total cholesterol, triglyceride, LDL-C, VFA, and remnant cholesterol and lower levels of HDL-C. Current smokers, heavy drinkers, and DM were also more prevalent in quartile 4 than in quartile 1.

**Table 1:** Baseline characteristics of study participants according to the TyG index quartiles in the cross-sectional analysis.

	TyG index				
	Quartile 1 *n* = 132	Quartile 2 *n* = 133	Quartile 3 *n* = 133	Quartile 4 *n* = 132	*p*
Age, years	51.00 (47.00–56.00)	53.00 (47.00–57.00)	55.00 (48.50–58.00)	55.00 (48.00–59.00)	0.014
Sex, *n* (%)					<0.001
Male	39 (29.5)	36 (27.1)	43 (32.3)	66 (50.0)	
Female	93 (70.5)	97 (72.9)	90 (67.7)	66 (50.0)	
BMI (kg/m^2^)	21.03 (19.98–22.30)	21.57 (20.49–22.87)	22.32 (21.05–23.23)	22.59 (21.13–23.20)	<0.001
Waist circumference (cm)	70.25 (68.00–75.00)	72.00 (68.00–76.00)	74.50 (70.00–79.00)	78.75 (72.50–82.00)	<0.001
SBP (mm Hg)	118.00 (110.00–124.75)	120.00 (110.00–130.00)	120.00 (110.00–134.00)	120.00 (112.00–138.00)	<0.001
DBP (mm Hg)	80.00 (70.00–80.00)	80.00 (70.00–81.50)	80.00 (70.00–86.00)	80.00 (76.50–90.00)	<0.001
FBG (mg/dL)	82.00 (77.00–88.00)	84.00 (79.00–90.00)	90.00 (83.00–98.00)	92.00 (84.00–108.00)	<0.001
Total cholesterol (mg/dL)	198.00 (172.00–218.25)	208.00 (184.00–230.00)	220.00 (192.00–250.50)	234.00 (206.00–258.75)	<0.001
HDL-C (mg/dL)	65.00 (56.25–76.00)	61.00 (55.00–69.50)	55.00 (49.00–62.00)	50.00 (40.25–55.00)	<0.001
LDL-C (mg/dL)	85.00 (70.50–94.00)	89.00 (77.00–101.00)	97.00 (82.50–111.00)	99.00 (81.00–113.75)	<0.001
Triglyceride (mg/dL)	62.50 (52.00–70.00)	93.00 (84.50–103.00)	122.00 (111.00–138.50)	192.00 (164.00–235.00)	<0.001
eGFR (mL/min per 1·73 m^2^)	103.32 (91.14–109.28)	104.11 (89.91–109.96)	102.27 (88.24–109.81)	102.92 (96.06–115.59)	0.248
VFA (cm^2^)	43.46 (29.72–61.63)	51.23 (34.89–66.09)	61.42 (45.18–78.56)	73.63 (57.67–107.65)	<0.001
SFA (cm^2^)	137.15 (104.65–175.65)	139.60 (106.55–182.20)	144.70 (109.20–183.80)	127.15 (107.10–158.38)	0.473
Body fat content, (%)	25.90 (21.85–28.50)	27.40 (23.15–30.75)	27.00 (23.55–31.10)	26.60 (23.60–30.75)	0.051
Remnant cholesterol (mg/dL)	46.00 (37.25–57.00)	57.00 (49.00–65.50)	67.00 (58.00–79.50)	81.50 (68.25–98.75)	<0.001
TyG index	7.85 (7.69–7.97)	8.27 (8.17–8.36)	8.64 (8.55–8.72)	9.12 (8.95–9.43)	<0.001
MONW, *n* (%)	8 (6.1)	10 (7.5)	33 (24.8)	102 (77.3)	<0.001
Smoking status, *n* (%)					<0.001
Non-smoker	105 (79.5)	104 (78.2)	104 (78.2)	78 (59.1)	
Ex-smoker	5 (3.8)	4 (3.0)	2 (1.5)	8 (6.1)	
Current smoker	22 (16.7)	25 (18.8)	27 (20.3)	46 (34.8)	
Alcohol consumption, *n* (%)					0.016
None	89 (67.4)	83 (62.4)	80 (60.2)	65 (49.2)	
Mild	22 (16.7)	27 (20.3)	26 (19.5)	33 (25.0)	
Heavy	21 (15.9)	23 (17.3)	27 (20.3)	34 (25.8)	
Hypertension, *n* (%)	13 (9.8)	26 (19.5)	38 (28.6)	34 (25.8)	0.117
DM, *n* (%)	3 (2.3)	2 (1.5)	19 (14.3)	22 (16.7)	<0.001
Physical activity, *n* (%)					0.202
Low	21 (15.9)	28 (21.1)	23 (17.3)	33 (25.0)	
Moderate	68 (51.5)	66 (49.6)	68 (51.1)	64 (48.5)	
High	43 (32.6)	39 (29.3)	42 (31.6)	35 (26.5)	

TyG index, triglyceride-glucose index; BMI, body mass index; SBP, systolic blood pressure; DBP, diastolic blood pressure; FBG, fasting blood glucose; HDL-C, high-density lipoprotein cholesterol; LDL-C, low-density lipoprotein cholesterol; eGFR, estimated glomerular filtration rate; VFA, visceral fat area; SFA, subcutaneous fat area; MONW, metabolically obese, normal weight; DM, diabetes mellitus.

**Figure 2:**
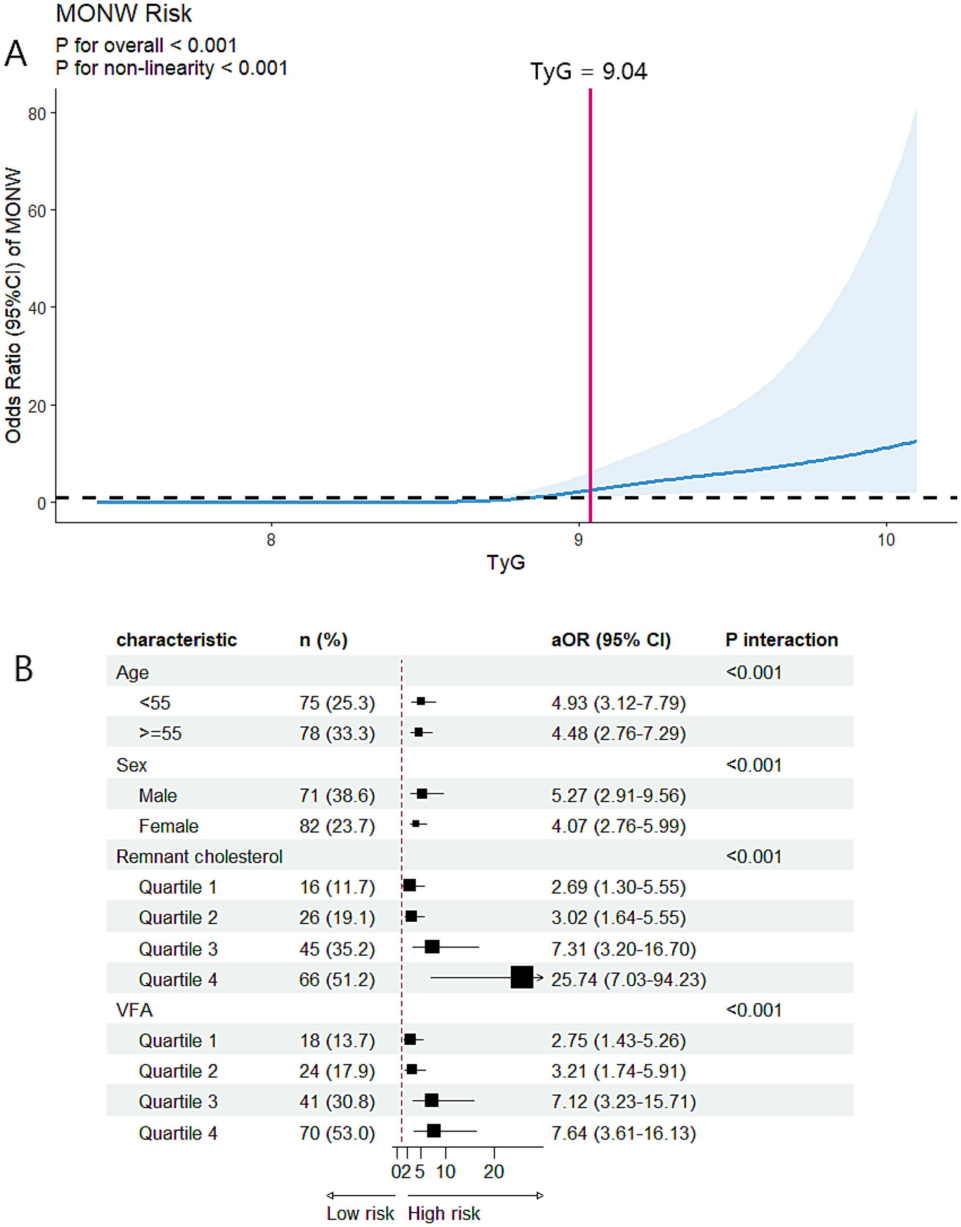
Associations between the TyG index and MONW in the cross-sectional analysis. **(A)** Restricted cubic spline regression analysis of the TyG index with MONW. The model was conducted with 5 knots at 5th, 35th, 50th, 65th, and 95th centiles of the TyG index (reference is the 5th centile). The ORs (95% CI) were adjusted for age, sex, BMI, waist circumference, smoking status, alcohol consumption, physical activity, hypertension, and DM. Solid lines indicate ORs, and shadow shapes indicate 95% CIs. **(B)** Subgroup analysis of the association between the TyG index and MONW. The aORs (95% CI) were estimated using logistic regression models adjusted for age, sex, BMI, waist circumference, smoking status, alcohol consumption, physical activity, hypertension, and DM. *p* interaction <0.05 indicated that the association between the TyG index and MONW differed significantly across subgroups. MONW, metabolically obese, normal weight; TyG index, triglyceride-glucose index; BMI, body mass index; DM, diabetes mellitus; aOR, adjusted odds ratio; CI, confidence interval; VFA, visceral fat area.

The prevalence of MONW progressively increased with the higher TyG index quartiles (6.1, 7.5, 24.8, and 77.3%, respectively). An incrementally higher risk of MONW was observed with the increasing TyG index quartiles compared with quartile 1 in all models (*p* trend <0.001). In the fully adjusted model (model 3), the adjusted OR in the TyG index quartile 4 was 31.81 (95% CI 11.47–88.20) for MONW (Table 2). The RCS model revealed a J-shaped association between the TyG index and risk of MONW (*p* for non-linearity <0.001), with a significant MONW risk for TyG >9.04 (Figure 2A). Subgroup analyses and interaction testing indicated the association between the TyG index and MONW differed significantly across age, sex, remnant cholesterol, and VFA subgroups and an incrementally higher MONW risk with higher remnant cholesterol and VFA quartiles (Figure 2B).

**Table 2:** Risk of MONW across the TyG index quartiles in the cross-sectional analysis.

	*n* (%)	OR (95% CI)	*p*	*p* trend	OR (95% CI)	*p*	*p* trend	OR (95% CI)	*p*	*p* trend
	Model 1				Model 2			Model 3		
Quartile 1	8 (6.1)	Ref			Ref			Ref		
Quartile 2	10 (7.5)	1.26 (0.48–3.30)	0.638		1.24 (0.47–3.25)	0.667		0.97 (0.34–2.75)	0.958	
Quartile 3	33 (24.8)	5.12 (2.26–11.57)	<0.001		4.78 (2.10–10.86)	<0.001		2.41 (0.94–6.21)	0.069	
Quartile 4	102 (77.3)	52.70 (23.15–119.98)	<0.001		48.25 (21.10–110.34)	<0.001		31.81 (11.47–88.20)	<0.001	
Overall	153 (28.9)	4.90 (3.68–6.51)		<0.001	4.71 (3.54–6.28)		<0.001	4.02 (2.82–5.73)		<0.001

Model 1: unadjusted.

Model 2: adjusted for age and sex.

Model 3: adjusted for age, sex, BMI, waist circumference, VFA, body fat content, remnant cholesterol, smoking status, alcohol consumption, physical activity, hypertension, and DM.

*p* trend <0.05 indicated the higher TyG index, the greater risk of MONW.

MONW, metabolically obese, normal weight; TyG index, triglyceride-glucose index; OR, odds ratio; CI, confidence interval; BMI, body mass index; VFA, visceral fat area; DM, diabetes mellitus.

### Follow-up analysis

A total of 253 eligible participants, including 82 male participants and 171 female participants, were included in the final follow-up analysis. Baseline participant characteristics stratified by the baseline TyG index quartiles are presented in Table 3 and were similar to the characteristics of the participants in the baseline cross-sectional analysis.

**Table 3:** Baseline characteristics of study participants according to the TyG index quartiles in the follow-up analysis.

	Baseline TyG index				
	Quartile 1 *n* = 63	Quartile 2 *n* = 63	Quartile 3 *n* = 64	Quartile 4 *n* = 63	*p*
Age, years	50.00 (47.00–54.00)	53.00 (47.00–57.00)	55.00 (50.25–58.00)	55.00 (50.00–59.00)	0.006
Sex, *n* (%)					0.011
Male	18 (28.6)	15 (23.8)	18 (28.1)	31 (49.2)	
Female	45 (71.4)	48 (76.2)	46 (71.9)	32 (50.8)	
BMI (kg/m^2^)	20.85 (19.87–21.94)	21.21 (20.49–22.44)	21.97 (20.98–22.89)	22.18 (21.12–22.99)	<0.001
Waist circumference (cm)	70.00 (67.00–75.00)	72.00 (68.00–74.50)	73.00 (69.25–78.75)	78.00 (73.50–82.00)	<0.001
SBP (mm Hg)	118.00 (110.00–124.00)	120.00 (105.00–130.00)	120.00 (112.00–130.00)	120.00 (118.00–138.00)	0.006
DBP (mm Hg)	80.00 (70.00–80.00)	80.00 (70.00–84.00)	80.00 (72.00–87.50)	80.00 (78.00–90.00)	0.033
FBG (mg/dL)	83.00 (79.00–89.00)	85.00 (80.00–90.00)	90.50 (83.00–98.75)	93.00 (84.00–112.00)	<0.001
Total cholesterol (mg/dL)	198.00 (168.00–216.00)	202.00 (183.00–224.00)	226.00 (196.25–254.00)	227.00 (206.00–248.00)	<0.001
HDL-C (mg/dL)	65.00 (57.00–76.00)	62.00 (51.00–71.00)	56.50 (50.00–62.00)	50.00 (40.00–54.00)	<0.001
LDL-C (mg/dL)	84.00 (63.00–93.00)	88.00 (76.00–101.00)	99.50 (88.25–111.75)	94.00 (78.00–109.00)	<0.001
Triglyceride (mg/dL)	60.00 (51.00–67.00)	87.00 (77.00–100.00)	115.50 (109.00–130.50)	193.00 (154.00–235.00)	<0.001
eGFR (mL/min per 1·73 m^2^)	102.56 (91.06–107.77)	106.21 (92.49–113.23)	99.34 (86.67–107.08)	101.98 (95.77–116.60)	0.126
VFA (cm^2^)	41.40 (28.36–54.95)	53.30 (35.87–71.72)	60.08 (34.14–77.54)	73.00 (58.94–105.80)	<0.001
SFA (cm^2^)	141.50 (102.90–180.30)	139.60 (112.30–165.20)	146.05 (107.20–183.03)	119.60 (102.10–158.40)	0.647
Body fat content, (%)	25.90 (21.70–28.00)	27.80 (24.00–30.90)	27.30 (22.90–30.95)	26.70 (24.10–30.90)	0.024
Remnant cholesterol (mg/dL)	43.00 (33.00–58.00)	54.00 (45.00–63.00)	67.50 (59.00–79.00)	80.00 (67.00–98.00)	<0.001
Baseline TyG index	7.84 (7.63–7.92)	8.23 (8.13–8.33)	8.59 (8.48–8.68)	9.10 (8.90–9.49)	<0.001
MNNW-MNNW, *n* (%)	51 (81.0)	42 (66.7)	27 (42.2)	8 (12.7)	<0.001
MNNW-MONW, *n* (%)	10 (15.9)	14 (22.2)	24 (37.5)	9 (14.3)	0.007
MONW-MONW, *n* (%)	1 (1.6)	6 (9.5)	11 (17.2)	39 (61.9)	<0.001
MONW-MNNW, *n* (%)	1 (1.6)	1 (1.6)	2 (3.1)	7 (11.1)	0.009
Smoking status, n (%)					0.020
Non-smoker	50 (79.4)	53 (84.1)	54 (84.4)	39 (61.9)	
Ex-smoker	2 (3.2)	1 (1.6)	0 (0.0)	5 (7.9)	
Current smoker	11 (17.5)	9 (14.3)	10 (15.6)	19 (30.2)	
Alcohol consumption, *n* (%)					0.698
None	43 (68.3)	42 (66.7)	41 (64.1)	35 (55.6)	
Mild	9 (14.3)	11 (17.5)	13 (20.3)	12 (19.0)	
Heavy	11 (17.5)	11 (15.9)	22 (34.4)	16 (25.4)	
Hypertension, *n* (%)	4 (6.3)	16 (25.4)	38 (28.6)	21 (33.3)	0.001
DM, *n* (%)	0 (0.0)	4 (6.3)	7 (10.9)	11 (17.5)	0.005
Physical activity, *n* (%)					0.264
Low	8 (12.7)	17 (27.0)	9 (14.1)	12 (19.0)	
Moderate	32 (50.8)	30 (47.6)	38 (59.4)	29 (46.0)	
High	23 (36.5)	16 (25.4)	17 (26.6)	22 (34.9)	

TyG index, triglyceride-glucose index; BMI, body mass index; SBP, systolic blood pressure; DBP, diastolic blood pressure; FBG, fasting blood glucose; HDL-C, high-density lipoprotein cholesterol; LDL-C, low-density lipoprotein cholesterol; eGFR, estimated glomerular filtration rate; VFA, visceral fat area; SFA, subcutaneous fat area; MNNW, metabolically normal, normal weight; MONW, metabolically obese, normal weight; DM, diabetes mellitus.

**Figure 3:**
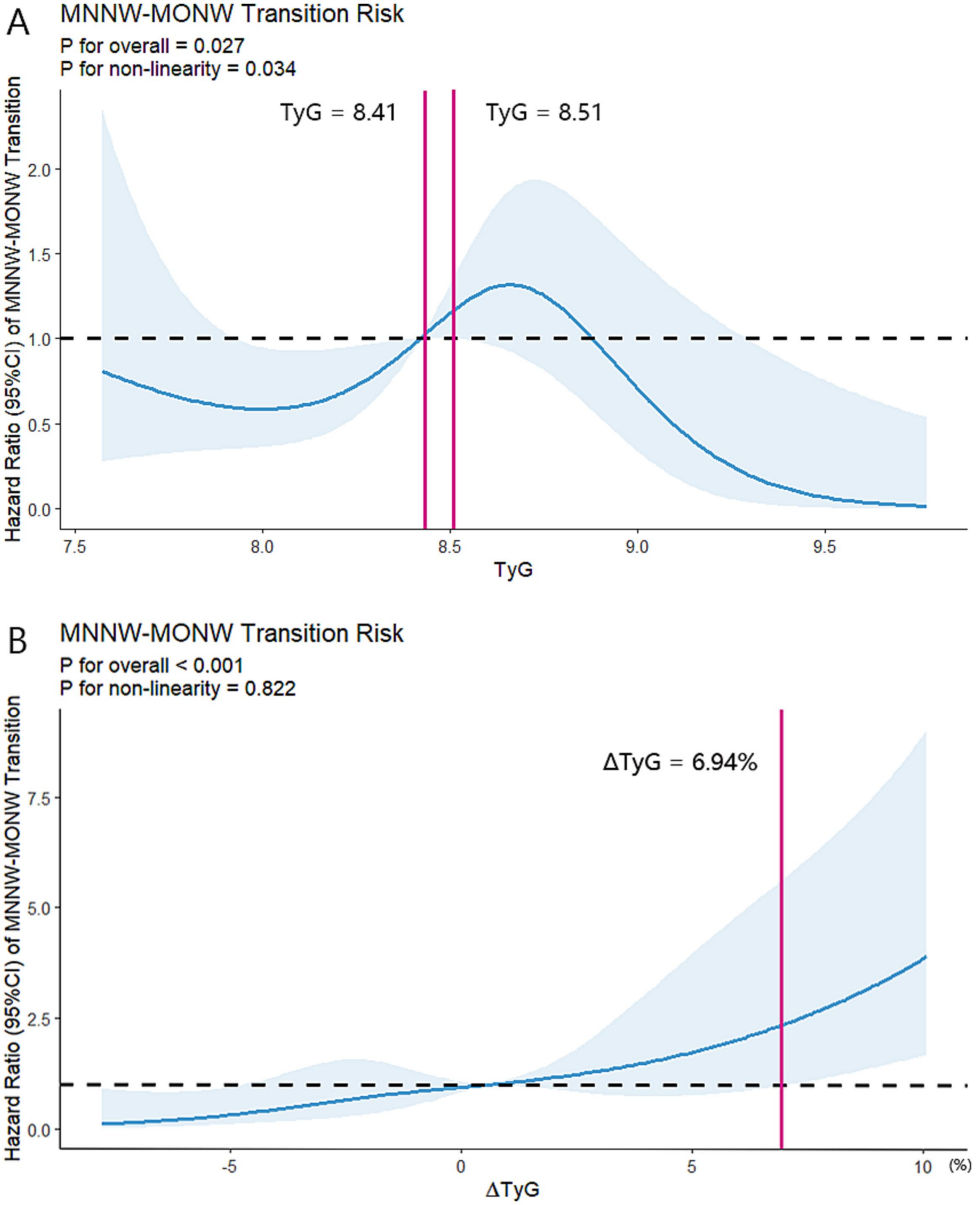
Restricted cubic spline regression analyses of two indices with MNNW-MONW transition in the follow-up analysis. **(A)** Restricted cubic spline regression analysis of the TyG index with MNNW-MONW transition. **(B)** Restricted cubic spline regression analysis of the ∆TyG index with MNNW-MONW transition. Two models were both conducted with 5 knots at 5th, 35th, 50th, 65th, and 95th centiles of each individual index (reference is the 5th centile). The HRs (95% CI) were adjusted for age, sex, BMI, waist circumference, smoking status, alcohol consumption, physical activity, hypertension, and DM. Solid lines indicate HRs, and shadow shapes indicate 95% CIs. MNNW, metabolically normal, normal weight; MONW, metabolically obese normal weight; TyG index, triglyceride-glucose index; HR, hazard ratio; CI, confidence interval; BMI, body mass index; DM, diabetes mellitus.

Furthermore, during a median follow-up of 5.0 (3.0–5.0) years, MNNW-MNNW, MNNW-MONW, MONW-MONW, and MONW-MNNW transitions occurred in 128 (50.6%), 57 (22.5%), 57 (22.5%), and 11 (4.3%) participants, respectively. The proportions of MONW-MONW and MONW-MNNW transition progressively increased with the increasing TyG index quartiles; however, the proportion of MNNW-MNNW progressively decreased with the increasing TyG index quartiles. Furthermore, the association between the TyG index and MNNW-MONW transition was not linear, with the highest MNNW-MONW transition occurring in the TyG index quartile 3 and the lowest occurring in quartile 4. In the fully adjusted model (model 3), the adjusted HR in the TyG index quartile 3 was 2.35 (95% CI 1.02–5.41) for MNNW-MONW transition (Table 4). In contrast, a positive linear association between the ∆TyG index quartiles and MNNW-MONW transition was observed in all models (*p* trend = 0.001), and in the fully adjusted model (model 3), the adjusted HR in the ∆TyG index quartile 4 was 3.60 (1.48–8.79) for the transition of MNNW-MONW (Table 4). In the RCS model, there was an n-shaped association between the TyG index and risk of MNNW-MONW transition (*p* for non-linearity = 0.034), with a significant MNNW-MONW transition risk for baseline TyG index between 8.41 and 8.51 (Figure 3A). Consistent with the results of the Cox proportional hazards regression model to fit the association between the ∆TyG index and MNNW-MONW transition, the RCS model also showed a linear association (*p* for non-linearity = 0.822), with a significant MNNW-MONW transition risk for ∆TyG index >6.94% (Figure 3B).

**Table 4:** Risk of MNNW-MONW transition across the TyG index and ∆TyG index quartiles in the follow-up analysis.

	*n* (%)	HR (95% CI)	*p*	*p* trend	HR (95% CI)	*p*	*p* trend	HR (95% CI)	*p*	*p* trend
	Model 1				Model 2			Model 3		
TyG index										
Quartile 1	10 (15.9)	Ref.			Ref.			Ref.		
Quartile 2	14 (22.2)	1.44 (0.64–3.24)	0.379		1.48 (0.66–3.33)	0.347		1.49 (0.638–3.50)	0.355	
Quartile 3	24 (37.5)	2.51 (1.20–5.25)	0.015		2.71 (1.28–5.75)	0.009		2.35 (1.02–5.41)	0.045	
Quartile 4	9 (14.3)	0.99 (0.40–2.44)	0.980		1.11 (0.44–2.79)	0.830		0.94 (0.31–2.84)	0.906	
Overall	57 (22.5)	1.09 (0.86–1.37)		0.506	1.13 (0.89–1.44)		0.325	1.09 (0.80–1.48)		0.602
∆TyG index										
Quartile 1	8 (12.7)	Ref.			Ref.			Ref.		
Quartile 2	10 (15.6)	1.21 (0.48–3.06)	0.689		1.18 (0.46–3.00)	0.732		1.33 (0.51–3.46)	0.561	
Quartile 3	18 (25.8)	2.27 (0.99–5.22)	0.054		2.21 (0.96–5.11)	0.063		2.90 (1.17–7.17)	0.022	
Quartile 4	21 (33.3)	2.60 (1.15–5.86)	0.022		2.51 (1.11–5.69)	0.027		3.60 (1.48–8.79)	0.005	
Overall	57 (22.5)	1.41 (1.10–1.80)		0.006	1.40 (1.09–1.78)		0.008	1.57 (1.20–2.07)		0.001

Model 1: unadjusted.

Model 2: adjusted for age and sex.

Model 3: adjusted for age, sex, BMI, waist circumference, VFA, body fat content, remnant cholesterol, smoking status, alcohol consumption, physical activity, hypertension, and DM.

*p* trend <0.05 indicated the higher TyG index and ∆TyG index, the greater risk of MNNW-MONW transition.

MNNW, metabolically normal, normal weight; MONW, metabolically obese normal weight; TyG index, triglyceride-glucose index; HR, hazard ratio; CI, confidence interval; BMI, body mass index; VFA, visceral fat area; DM, diabetes mellitus.

**Figure 4:**
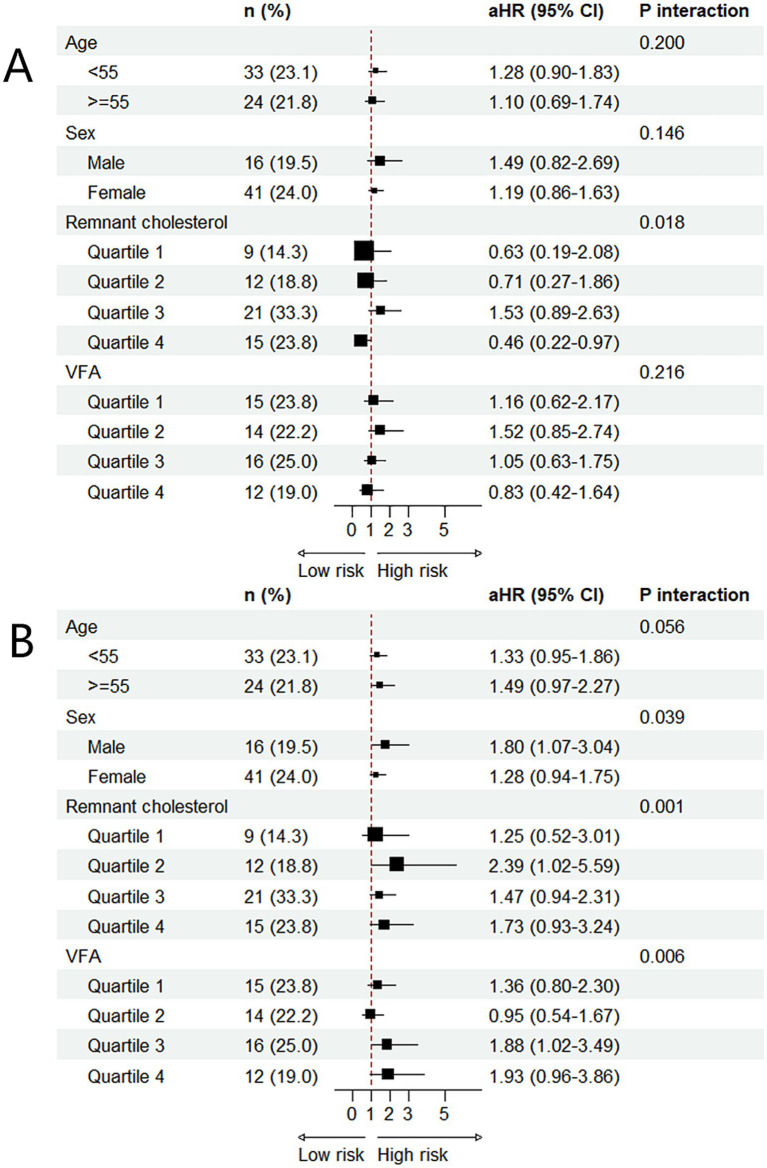
Subgroup analysis of the associations between two indices and MNNW-MONW transition in the follow-up analysis. **(A)** The association between the TyG index and MNNW-MONW transition. **(B)** The association between the ∆TyG index and MNNW-MONW transition. The aHRs (95% CI) were estimated using Cox regression models adjusted for age, sex, BMI, waist circumference, smoking status, alcohol consumption, physical activity, hypertension, and DM. *p* interaction <0.05 indicated that associations among the TyG index, ∆TyG index, and MNNW-MONW transition differed significantly between subgroups. MNNW, metabolically normal, normal weight; MONW, metabolically obese normal weight; TyG index, triglyceride-glucose index; aHR, adjusted hazard ratio; CI, confidence interval; VFA, visceral fat area; BMI, body mass index; DM, diabetes mellitus.

Subgroup analyses and interaction testing showed that the association between the TyG index and MNNW-MONW transition differed significantly only between remnant cholesterol subgroups, and surprisingly, the higher TyG index was a protective factor for MNNW-MONW transition in the remnant cholesterol quartile 4 with the adjusted HR being 0.46 (0.22–0.97). Furthermore, the association between the ∆TyG index and MNNW-MONW transition differed significantly across sex, remnant cholesterol, and VFA subgroups, and there was a trend for an incrementally higher MNNW-MONW transition risk with higher VFA quartiles (Figure 4).

### Sensitivity analysis

Sensitivity analysis excluding participants with MONW at baseline also revealed positive linear associations between the TyG index quartiles, ∆TyG index quartiles, and the transition of MNNW-MONW: the increasing TyG index quartiles and ∆TyG index quartiles were associated with a progressively higher risk of MNNW-MONW transition compared with quartile 1 (*p* trend <0.05). The results of sensitivity analysis for the association between the ∆TyG index quartiles and the transition of MNNW-MONW were similar to those in the follow-up analysis (Supplementary Table 1). In addition, in the subgroup analyses and interaction testing, we observed the association between the TyG index and the transition of MNNW-MONW differed significantly across age, sex, remnant cholesterol, and VFA subgroups; however, the association between the ∆TyG index and MNNW-MONW transition differed significantly only between remnant cholesterol and VFA subgroups, which was also similar to the results of the follow-up analysis. Furthermore, the increased risk of MNNW-MONW transition associated with either the TyG index or ∆TyG index was more pronounced in VFA quartile 4 in the subgroup analyses and interaction testing (Supplementary Figure 1).

## Discussion

In this population-based cohort study, we found that the higher TyG index was significantly associated with higher risk of MONW in the cross-sectional study; even more importantly, the higher TyG index and ∆TyG index were significantly associated with higher risk of MNNW-MONW transition in the 5-year follow-up analysis, and these relationships were further strengthened by larger VFA. These findings suggested the TyG and ΔTyG index were sensitive and cost-effective screening tests to monitor and mitigate the risk of MONW-MONW and MNNW-MONW transition and the accompanying CVD risk, especially in people with large VFA. In addition, the predictive performance of the ∆TyG index was better and more stable than the TyG index when monitoring the risk of MNNW-MONW transition, indicating dynamic changes of the TyG index should be monitored regularly in clinical practice for timely recognition of MNNW-MONW transition.

Emerging evidence supported the prediction performance of the TyG index in various chronic metabolic diseases, including type 2 diabetes and CVD (7–10). Previously, several cross-sectional studies also supported the association between the TyG index and MONW incidence (8, 24, 25); however, no studies have evaluated the effects of the TyG index and its changes in predicting MNNW-MONW transition from the long-term metabolic abnormality perspective. Therefore, our study is of great importance. Consistently, we also found that the TyG index is correlated with MOMN in our cross-sectional analysis (8, 24, 25). However, we were the first to report the positive relationship between the ∆TyG index and MNNW-MONW transition in a 5-year follow-up investigation. Although the mechanism underlying the TyG index and its changes in relation to MOMN and MNNW-MONW transition remain unclear, it may be partially elucidated from the perspective of insulin resistance, which is currently recognized as the key etiology of MONW (2, 4). Previous studies have reported the significance of the TyG index in assessing insulin resistance as compared with hyperinsulinemic-euglycemic clamp, as well as the role of the TyG index in evaluating whole-body insulin resistance and CVD prognosis (7–10). In addition, positive associations between components of MONW and the TyG index have also been reported (8). Furthermore, participants in our study were middle-aged and older adults who were more susceptible to visceral fat deposition and insulin resistance-related metabolic abnormalities. Therefore, simple indicators such as the TyG index, which reflects insulin resistance, are more practically significant for monitoring metabolic phenotype transitions, particularly for Asians even with a normal BMI (26, 27). Based on the findings regarding the relationship between the TyG index and MOMN, as well as the association between the TyG index, ∆TyG index, and MNNW-MONW transition, insulin resistance emerges as a pivotal factor for both MOMN and the metabolic phenotype conversion.

We revealed a significant interaction effect between sex, VFA and the TyG index, and ∆TyG index for the risk of MONW-MONW and MNNW-MONW transition by subgroup analyses and interaction testing. It has been reported that, although MONW individuals maintained a normal BMI, their fat distribution tended to accumulate in the truncal or visceral adipose tissue (11). The difference between sex may be partly attributed to a greater amount of visceral fat in male participants and more subcutaneous adipose tissue in female participants (28). Sex hormones also influence body fat distribution and adipocyte differentiation, thereby influencing the difference of MONW phenotype between genders (29). In addition, there were distinct adipose progenitor cells in visceral fat in male participants with elevated proliferation and adipogenesis activity during middle age, which partly contributed to visceral fat expansion of middle-aged male participants (30). In addition, several studies have reported a positive correlation between the TyG index and visceral fat deposition (31–33). Excess visceral fat deposition is linked to the development of insulin resistance and metabolic impairment (34, 35). The increase in visceral adipose tissue leads to the excessive release of pro-inflammatory cytokines and is prone to lipolysis, which in turn accumulates free fatty acids in the liver and skeletal muscles. In the liver, excess free fatty acids contribute to increased very-low-density lipoprotein biosynthesis and reduced degradation, which eventually translate into an increase in low-density lipoprotein particles with high atherogenic property and an increase in the concentration of triglyceride in the blood plasma disturbing the insulin pathways. On the other hand, the accumulation of free fatty acids in skeletal muscles adversely affects the activity of insulin, leading to reduced glucose utilization in skeletal muscles. Thus, the TyG index reflects the combined effects of increased hepatic triglyceride synthesis and decreased glucose utilization in skeletal muscle, both resulting from visceral fat deposition. From this perspective, the relationships between the TyG index and MONW and between the TyG index, ∆TyG index, and MNMN-MOMN transition are closely linked to visceral fat.

Notably, the associations between the TyG index quartiles and MNNW-MONW transition were not consistent in the follow-up analysis or sensitivity analysis, which may be due to the significant difference in the proportions of MNNW at baseline among the TyG index quartiles. Since only 17 (26.9%) participants with MNNW were at baseline in the TyG index quartile 4 in the follow-up analysis, there was a limited ceiling of proportion of MNNW-MONW transition in quartile 4. On the other hand, participants with MONW at baseline may be more likely to gain much BMI, exacerbate metabolic dysfunction, and not meet the purpose of the study. It is also easy to understand MONW-MNNW transition increases with the increasing TyG index quartiles because the proportion of MONW increases with the increasing quartiles. In addition, the seemingly contradictory point the TyG index in quartile 4 was a protective factor for MNNW-MONW transition in the remnant cholesterol quartile 4 might be owing to the lowest proportion of MNNW in the TyG index quartile 4. After excluding participants with pre-existing MONW, the ceiling of the proportion of MNNW-MONW transition was equal in 4 quartiles; therefore, the association between the TyG index and MNNW-MONW transition can be rationally presented under this condition. Therefore, we have reasons to believe that there is a positive linear association between the TyG index and MNNW-MONW transition. Nevertheless, the associations between the ∆TyG index quartiles and MNNW-MONW transition were consistent in the follow-up analysis and sensitivity analysis, which suggested the ∆TyG index may be more stable and reliable for indicating the risk of MNNW-MONW transition as compared to the TyG index.

In addition, it is important to acknowledge that definitions and diagnostic criteria for DM and hypertension vary across different guidelines, which may influence MONW identification and study outcomes. In the current study, we adopted Chinese guidelines for DM and hypertension, which are suitable for Chinese population but have some variations compared to other guidelines, such as European and American guidelines. These differences can impact the prevalence of MONW-MONW and MNNW-MONW transition and the strength of associations among the TyG index, ∆TyG index, and MONW-MONW and MNNW-MONW transition, which limited the comparability and generalizability of the conclusions.

Several limitations of this study should be acknowledged. First, definitions and diagnostic criteria for DM, overweight/obesity, and hypertension vary across different guidelines, and we adopted definitions applicable to the Chinese demographic in the current study, which may influence the prevalence of MONW-MONW and MNNW-MONW transition and limit the comparability and generalizability of the conclusions to other ethnic groups. Second, although the inclusion population in the follow-up analysis can represent the overall population, exclusion of participants with non-normal BMI levels may result in the dilution of the predictive power of the TyG and ∆TyG index for severe metabolic abnormalities (overweight/obesity/underweight) and exacerbate the inherent bias of observational studies, such as selection bias, and future studies need to explore the role of the TyG and ∆TyG index in a larger field of metabolic abnormalities with a larger sample. Third, some inherent and potential bias of the observational study should be considered, such as residual confounding, reverse causation, selection bias, and recall bias for several covariates and self-reported variables (smoking status, alcohol consumption, and physical activity). Fourth, the small amount of the study sample size and the study population located in a specific region limited the generalizability of the conclusions. Fifth, the individuals in the study were predominantly middle-aged and older adults, which limited the extrapolation of the results; therefore, additional investigations are needed to extrapolate the results to other age and ethnic groups.

## Conclusion

The elevated TyG index and ∆TyG index were associated with higher risks of MONW-MONW and MNNW-MONW transition, and the ∆TyG index may be a better biomarker for indicating the risk of MNNW-MONW transition. The risks of MONW-MONW and MNNW-MONW transition associated with the higher TyG index and ∆TyG index were strengthened by VFA.

## Data Availability

The raw data supporting the conclusions of this article will be made available by the authors, without undue reservation.

## Footnotes

^1^ http://www.R-project.org

## Glossary

2hPG: 2-h post-load plasma glucose
ANOVA: One-way analysis of variance
BMI: Body mass index
CIs: Confidence intervals
CVD: Cardiovascular disease
DBP: Diastolic blood pressure
DM: Diabetes mellitus
FBG: Fasting blood glucose
HDL-C: High-density lipoprotein cholesterol
HRs: Hazard ratios
IPAQ-SF: International Physical Activity Questionnaire Short Form
LDL-C: Low-density lipoprotein cholesterol
MNNW: Metabolically normal, normal weight
MONW: Metabolically obese normal weight
MRI: Magnetic resonance imaging
OGTT: Oral glucose tolerance test
ORs: Odds ratios
SBP: Systolic blood pressure
SD: Standard deviation
SFA: Subcutaneous fat area
TyG index: Triglyceride-glucose index
VFA: Visceral fat area
RCS: Restricted cubic spline
