# Supplementary material for: Relationships among the triglyceride-glucose index, its changes, and the development of metabolically obese normal weight are strengthened by increased visceral fat area
Source: Front Nutr. 2025 Sep 16;12:1642725. doi: 10.3389/fnut.2025.1642725 (PMC12479333; doi:10.3389/fnut.2025.1642725)
Supplement: Supplementary file 1 [file Supplementary_file_1.docx]

**Supplementary Materials**

**Supplementary Figures**


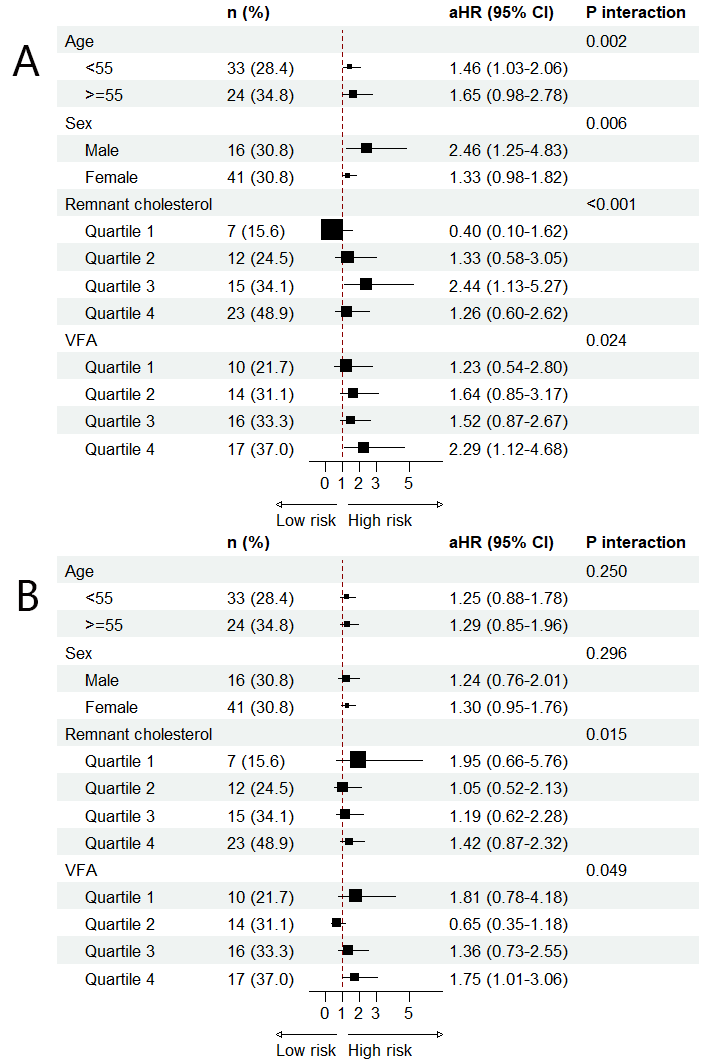


**Supplementary Figure 1** Subgroup analysis of the associations between two indices and MNNW-MONW transition in sensitivity analysis

1. The association between the TyG index and MNNW-MONW transition. (B) The association between the ∆TyG index and MNNW-MONW transition. The aHRs (95% CI) were estimated using Cox regression models adjusted for age, sex, BMI, waist circumference, smoking status, alcohol consumption, physical activity, hypertension and DM. *P* interaction <0.05 indicated that associations between the TyG index, ∆TyG index and MNNW-MONW transition differed significantly across subgroups.

MNNW = metabolically normal, normal weight. MONW = metabolically obese, normal weight. TyG index = triglyceride-glucose index. aHR = adjusted hazard ratio. CI = confidence interval. VFA = visceral fat area. BMI = body-mass index. DM = diabetes mellitus.

**Supplementary Tables**

**Supplementary** **Table 1** Risk of MNNW-MONW transition across two indices quartiles in sensitivity analysis

|  | n (%) | aHR (95% CI) | *P* | *P* trend |
| --- | --- | --- | --- | --- |
| TyG index |  |  |  |  |
| Quartile 1 | 9 (19.6) | Ref |  |  |
| Quartile 2 | 10 (21.7) | 1.06 (0.42-2.65) | 0.905 |  |
| Quartile 3 | 10 (22.2) | 1.10 (0.43-2.82) | 0.848 |  |
| Quartile 4 | 28 (58.3) | 3.24 (1.42-7.41) | 0.004 |  |
| Overall | 57 (30.8) | 1.55 (1.18-2.05) |  | 0.002 |
| ∆TyG index |  |  |  |  |
| Quartile 1 | 11 (23.9) | Ref |  |  |
| Quartile 2 | 11 (23.9) | 1.07 (0.44-2.56) | 0.883 |  |
| Quartile 3 | 16 (34.0) | 1.58 (0.71-3.53) | 0.267 |  |
| Quartile 4 | 19 (41.3) | 2.53 (1.13-5.67) | 0.024 |  |
| Overall | 57 (30.8) | 1.39 (1.07-1.80) |  | 0.012 |

The aHRs (95% CI) were estimated using Cox regression models adjusted for age, sex, BMI, waist circumference, VFA, body fat content, remnant cholesterol, smoking status, alcohol consumption, physical activity, hypertension and DM. *P* trend <0.05 indicated the higher TyG index and ∆TyG index, the greater risk of MNNW-MONW.

MNNW = metabolically normal, normal weight. MONW = metabolically obese, normal weight. TyG index = triglyceride-glucose index. aHR = adjusted hazard ratio. CI = confidence interval. BMI = body-mass index. VFA = visceral fat area. DM = diabetes mellitus.
